# Real-time feedback of air quality in children’s bedrooms reduces exposure to secondhand smoke

**DOI:** 10.18332/tpc/149908

**Published:** 2022-06-22

**Authors:** Vincent Berardi, Bradley N. Collins, Laura M. Glynn, Stephen J. Lepore, E. Melinda Mahabee-Gittens, Karen M. Wilson, Melbourne F. Hovell

**Affiliations:** 1 Department of Psychology, Crean College of Health and Behavioral Sciences, Chapman University, Orange, United States; 2 Department of Social and Behavioral Sciences, Temple University, Philadelphia, United States; 3 Cincinnati Children’s Hospital Medical Center, University of Cincinnati College of Medicine, Cincinnati, United States; 4 University of Rochester Medical Center, Rochester, United States; 5 San Diego State University Graduate School of Public Health, San Diego, United States

**Keywords:** secondhand smoke, air quality, youth, air monitoring, smoke-free environments, in-home smoking

## Abstract

**INTRODUCTION:**

Secondhand smoke (SHS) exposure creates health risks for non-smokers and is especially detrimental to children. This study evaluated whether immediate feedback in response to poor indoor air quality in children’s bedrooms can reduce the potential for SHS exposure, as measured by adherence to a World Health Organization (WHO) indoor air standard.

**METHODS:**

Homes that contained children and an adult who regularly smoked inside (n=298) had an air particle monitor installed in the child’s bedroom. These devices measured the concentration of particulate matter (PM2.5) for approximately three months and, for half of the participants, immediately provided aversive feedback in response to elevated PM2.5. Hierarchical linear models were fit to the data to assess whether the intervention increased the probability that: 1) a given day was below the WHO guideline for daily exposure, and 2) a household established and maintained a smoke-free home (SFH), operationalized as achieving 30 consecutive days below the WHO guideline. The intervention’s impact was calculated as group-by-time effects.

**RESULTS:**

The likelihood that a child’s bedroom met the WHO indoor air quality standard on a given day increased such that the baseline versus post-baseline odds ratio (OR) of maintaining indoor PM2.5 levels below the WHO guideline was 2.38 times larger for participants who received the intervention. Similarly, the baseline versus post-baseline OR associated with achieving an SFH was 3.49 times larger for participants in the intervention group.

**CONCLUSIONS:**

The real-time intervention successfully drove clinically meaningful changes in smoking behavior that mitigated indoor PM2.5 levels in children’s bedrooms and thereby reduced SHS exposure. These results demonstrate the effectiveness of targeting sensitive microenvironments by giving caregivers actionable information about children’s SHS risks. Future extensions should examine additional microenvironments and focus on identifying the potential for SHS exposure before it occurs.

## INTRODUCTION

Secondhand smoke (SHS) is responsible for >41000 deaths and $5.6 billion in lost productivity in the US each year^1^. Compared to adults, children are exceptionally vulnerable to the adverse health effects associated with SHS^2,3^, which include sudden infant death syndrome, acute respiratory infections, and increased asthma severity^1^. SHS can also sensitize children to nicotine, which may increase their risk of smoking in adolescence^4^. The home is the primary location where SHS exposure occurs^5^, and children’s bedrooms can be a particularly hazardous environment. Young children typically go to bed in the early evening^6^, approximately three hours before adults^7^, resulting in a period when children are sleeping while adults may smoke in the home. Previous studies have reported the presence of a ‘daily dip-evening incline’ class of smokers, with an elevated frequency of smoking later in the evening^8^. If children are sleeping while late-evening, in-home smoking occurs, SHS may infiltrate into their bedrooms and increase their exposure.

Parents and other adults may expose children to SHS because they believe that children are safe if SHS cannot be seen or smelled^9^. However, the smoke from one cigarette can persist in the air of multiple residential rooms for over two hours after a cigarette is extinguished^10^. Thus, even though children’s SHS exposure is greatest when they are present in the same room where a parent is smoking, SHS can permeate and persist in other environments such as children’s bedrooms, potentially without caregivers’ knowledge. Providing microenvironment feedback about SHS exposure may help caregivers understand the pervasiveness and scope of residential SHS exposure, attune them to its risks, and mobilize them to adhere to SHS reduction strategies^11,12^. With this goal in mind, the Project Fresh Air (PFA) study was recently completed^13,14^. This hybrid multiple baseline/randomized clinical trial deployed air particle quality monitors with real-time feedback mechanisms in multiple locations in the homes of smokers who lived with a child. The intervention was grounded in operant theory and objective (rather than self-reported) measures of SHS exposure.

The PFA study was novel in its technology infrastructure, intensity of longitudinal measures, and real-time intervention approach. Therefore, much of the reporting thus far has focused on design and protocols^13-15^. Consequently, statistical analyses have been broad and considered data from only a single location, the self-reported main smoking room. PFA’s effect on reducing air particle levels and the occurrence of smoking episodes has been quantified; yet outcomes have not yet been compared to health-based guidelines, which limits our ability to fully assess the benefits of the intervention. The current study aimed to characterize the effect of PFA intervention more specifically by: 1) examining data from environments where children slept, and 2) investigating PFA’s impact on outcomes associated with a World Health Organization (WHO) indoor air quality standard.

## METHODS

### Project Fresh Air design

Full details of the PFA design and sample have been published elsewhere^13,14^. Briefly, 298 predominately low-income, racially/ethnically diverse households in San Diego County, California, in which at least one adult smoker and a child under 14 years of age lived, were enrolled in the study. Dylos DC1700 (Riverside, CA) air particle monitors were installed in the room nearest to where most smoking occurred and in the child’s bedroom for approximately 90 days and recorded a measure of indoor air quality every 10 seconds. Homes were block randomized as pairs into one of two groups: 1) a measurement-only control condition, or 2) an intervention condition. Intervention homes were stratified into two phases: 1) a measurement-only baseline, and 2) a post-baseline period during which immediate feedback in the form of a persistent red/orange LED and an aversive tone was presented in response to elevated air particle measurements above 15000 counts of fine particulate matter (PM2.5), which is consistent with likely SHS exposure. This feedback was supplemented by periodic home visits during which PFA personnel reviewed printouts of recent air particle history and discussed strategies to either establish or maintain smoke-free homes (SFHs). The study was approved by the San Diego State University Institutional Review Board.

### Measures

*WHO daily PM2.5 guideline*

For each household, the daily mean counts of PM2.5 from the child’s room monitor was calculated and then converted to mass concentrations (μg/m^3^ ) according to previously-developed procedures^15^. The mass concentration was then compared to the WHO indoor air guideline of maintaining indoor PM2.5 levels below 25 μg/m^3^ over a one-day period^16^. On each day (29925 total days), the child’s bedroom was dichotomously coded as either exceeding (0) or below (1) the WHO guideline.

*Smoke-free home status*

We also constructed a measure that serves as a proxy for participants’ SFH implementation and maintenance over the 90-day assessment period. Homes with any instance of 30 consecutive days below the WHO guideline during the post-baseline phase were scored as having successfully established a monitor-verified SFH and were coded as 1, versus 0 (unsuccessful in establishing an SFH). This determination was also made for the baseline, but because the time spent in this phase was an average of 56% of that in the post-baseline phase, the baseline criterion was set to 0.56×30 = 17 days. The evaluation of a single block of 30/17 days below the guideline within this measure was intended to be strict enough to capture meaningful participant behavior, while flexible enough to account for particle sources beyond the control of smokers, e.g. vehicle exhaust infiltrating into the home.

### Statistical approach

Control homes did not receive an intervention, so the baseline/post-baseline delineation for each control home was assigned to that of its corresponding intervention home. For each of the four group/phase combinations, the mean PM2.5 concentration was calculated for all homes and all days.

To assess the effect of the intervention on the probability of being below the WHO guideline, the following random-intercept, hierarchical logistic regression model with an interaction term (Model 1) was fit to the data:

*w_i,j_= β_0_ + β_1_ g + β_2_ p + β_3_ g∙p + β_4i_,*

where *w_i,j_* is the WHO guideline status (0 vs 1) of individual *i* on day *j*, *g* is the control (0) versus intervention (1) group, *p* is the baseline (0) versus post-baseline (1) phase, and the *β* are regression coefficients, including the random intercept *β_4i_* for each participant.

For the SFH outcome (Model 2), *w_i,j_* represents the SFH status for individual *i* during phase *j*, where *j* is either baseline (0) or post-baseline (1). Since participants who were enrolled longer had a greater opportunity to meet the SFH criteria, we also controlled for the total number of days of enrollment. Only those homes with at least 17 baseline days and 30 post-baseline days (n=269) were included in this analysis.

## RESULTS

On average, households were enrolled in the baseline phase for 36.3 days (SD=14.2) and the post-baseline phase for 64.8 (26.6) days. Overall, 93.0% of all days were in adherence with the WHO indoor air daily guideline and 79.9%/79.1% of participants established a monitor-verified SFH in the baseline/post-baseline phases. The mean PM2.5 concentration (μg/m^3^) across all days in the four group/phase combinations were: control/baseline 11.6; control/post-baseline 9.9; intervention/baseline 12.3; and intervention/post-baseline 9.3.

The results of the two regression models are shown in Table 1. Model 1 indicates that the PFA intervention increased the likelihood that a child’s bedroom PM2.5 levels were below the WHO indoor air quality guideline on a given day, indicated by the statistically significant group-by-phase interaction term. Specifically, the odds ratio (OR) of WHO guideline achievement from baseline to post-baseline was 2.38 times larger for participants in the intervention group. Neither group nor phase main effects were statistically significant.

**Table 1:** Results of two regression models

*Models*	*OR*	*95% CI*	*p*
**Model 1**			
Group (*g*, Experimental = 1)	0.56	0.25–1.26	0.16
Phase (*p*, Post-baseline = 1)	1.18	0.98–1.42	0.09
Group × Phase (*g∙p*)	2.38	1.81–3.12	<0.001*
**Model 2**			
Group (*g*, Experimental = 1)	0.46	0.16–1.36	0.16
Phase (*p*, Post-baseline = 1)	0.51	0.23–1.13	0.10
Group × Phase (*g∙p*)	3.49	1.08–11.27	0.03
Total enrollment days^a^	1.69	1.06–2.70	0.04

OR: odds ratio. Model 1: is a day-level, hierarchical logistic regression model with below the WHO indoor air quality guideline (outcome = 1) as the dependent variable and participant serving as the random-intercept effect. Model 2: is a phase-level, hierarchical logistic regression model with the establishment of an SFH (SFH = 1) as the dependent variable and participant serving as the random-intercept effect.

a Standardized version of the variable was used to help with model convergence. All participants (n=298) were included in Model 1 and only those participants with at least 17/30 baseline/post-baseline measures (n=269) were included in Model 2.

Model 2 demonstrates that PFA also increased the likelihood that a home established and maintained an SFH. The significant group-by-phase interaction term indicates that the OR for having a SFH from baseline to post-baseline was 3.49 times larger for participants in the intervention group. The relationship between SFH and total enrollment days was also significant, such that a one standard deviation increase in total enrollment days was associated with 1.69 larger odds of establishing an SFH. Neither group nor phase main effects were statistically significant.

## DISCUSSION

This study extends the previous dissemination of PFA outcomes by examining data collected directly from children’s bedrooms and demonstrating reductions in health-based indoor air measures in accordance with a WHO guideline for daily PM2.5 exposure. We found that the intervention significantly reduced the probability of a given day exceeding the WHO guideline and that it also increased the probability of households establishing an SFH, operationalized as a 30-day block of consecutive days below the WHO guideline.

The focus of this analysis on the child’s bedroom is important since SHS is particularly insidious for children and has elevated risks when they are sleeping. Characteristics, such as higher breathing rates, immature lungs and underdeveloped immune systems make it difficult to filter toxins^2,3^ and children inhale a larger volume of air per body mass than adults^17^, which results in higher relative doses of inhalation-related exposure to SHS pollutants. While sleeping, playing, studying, or engaging in other activities in their bedroom, SHS may infiltrate into the environment without their caregivers’ knowledge. Since there is no safe level of SHS, improving caregivers’ awareness of the extent to which their residential smoking impacts their children’s bedroom environments could substantially influence efforts to mitigate SHS and create an SFH.

Our results indicate that PFA successfully changed parent’s residential indoor smoking behavior and reduced the probability of exceeding a WHO guideline for indoor air quality and PM2.5 exposure, which is consistent with other studies showing that feedback concerning indoor air quality can improve tobacco-related outcomes^18-21^. However, the PFA study is differentiated from the others by its immediate feedback characteristics, which is grounded in operant theory and provides caregivers with real-time, actionable information to protect their children’s health. The objective data generated by the air particle monitors are also a strength of the PFA approach, since it can be incorporated into other intervention modalities, such as supportive health education and counseling, which was done in PFA. Future extensions of PFA may use the data from multiple air monitors and other sensors to identify the potential for SHS exposure in children’s bedrooms before it occurs and present real-time suggestions for appropriate mitigation steps.

### Limitations

There are limitations to this study, and the PFA approach in general. The statistical analysis used the WHO’s daily guideline of 25 μg/m^3^ PM2.5 as a health threshold, but the WHO also has a 10 μg/m^3^ average annual guideline, which would be appropriate if data were collected for a longer duration and/or this study was not meant to expand upon previously assessed day-level outcomes^13-15^. PFA feedback was not based on the WHO criterion and there is no guarantee that participants observed the aversive stimuli provided in response to elevated PM2.5 levels. Outdoor air quality, which could affect indoor air PM2.5 concentrations, was not included in our models since this effect is expected to be similar across groups, thereby having a minimal impact on outcomes that compare control to intervention homes. Despite these shortcomings, our results indicate that the real-time sensing/feedback capabilities exemplified by PFA represent an opportunity to shift interventions towards being more widespread, robust, and theory-based, potentially making them more capable of improving public health.

## Data Availability

The data supporting this research are available from the authors on reasonable request.
